# Cider vinegar rules

**DOI:** 10.7554/eLife.40271

**Published:** 2018-08-24

**Authors:** Ronald L Calabrese

**Affiliations:** Department of BiologyEmory UniversityAtlantaUnited States

**Keywords:** olfaction, behavior, navigation, computation, chemotaxis, *D. melanogaster*

## Abstract

Experiments in wind tunnels have shed light on the rules that govern how flies respond when they detect odors.

**Related research article** Álvarez-Salvado E, Licata AM, Connor EG, McHugh MK, King BM, Stavropoulos N, Victor JD, Crimaldi JP, Nagel KI. 2018. Elementary sensory-motor transformations underlying olfactory navigation in walking fruit-flies. *eLife* **7**:e37815. doi: 10.7554/eLife.37815

Running through an unfamiliar area, the familiar odor of fast food wafts into your nostrils and you turn your head to see where it is coming from – even though you know that fast food is not good for your health. Flies respond in a similar way when they smell apple cider vinegar. But how are humans or flies able to locate the source of a scent or odor? The answer lies in a process called chemotaxis – the technical term for orienting to a scent or odor and pursuing or avoiding it.

Chemotaxis has been studied for decades. In *The Orientation of Animals*, first published in 1940, Gottfried Fraenkel and Donald Gunn defined many of the concepts and terms that are central to chemotaxis (Fraenkel and Gunn, 1961). In particular, they recognized that chemicals – unlike other stimuli such as gravity and light – have no inherent directional component and thus require different strategies for taxis.

In relatively still water or air, chemical gradients are diffusion limited and relatively stable: thus, by sampling the chemical over time, the organism can determine the structure of a chemical gradient. When confronted with such a gradient some species, such as *E. coli* (Berg, 2004) and *C. elegans* (Lockery, 2011), move in a straight line and then randomly change direction when the chemical cue becomes weaker.

*Drosophila* larvae, on the other hand, are more sophisticated and preferentially turn toward higher concentrations of the chemical cue. The ability of *Drosophila* larvae to detect very small decreases in the concentration of chemical cues, combined with the widespread availability of tools for the genetic manipulation of flies, has resulted in this species becoming an important model system for the study of chemotaxis (Gepner et al., 2015; Hernandez-Nunez et al., 2015; Schulze et al., 2015; Calabrese, 2015).

However, the air is not still in many environments, with air currents disrupting chemical gradients and odor plumes. Close to the ground or another surface, the air flow may be laminar (that is, not turbulent), and the structure of odor plumes is relatively stable. Under these conditions, the air flow provides directional information that helps flies find the source of the odor. For a flying insect, however, locating the source of an odor means navigating a turbulent odor plume where changes in odor concentration can be rapid and do not directly tell the insect if it is moving toward or away from a source.

The ability of moths to follow the odor plume of a sex pheromone has been extensively studied (see, for example, Baker, 1990). The moths fly upwind in the presence of the odor until they lose the scent, and then start flying across the wind; once the odor is encountered again, they resume flying upwind. Adult flying *Drosophila* employ a similar strategy, with the wind providing a directional cue that is not present in the temporal structure of the chemical signal (Bhandawat et al., 2010). Now, in eLife, Katherine Nagel of New York University (NYU) and co-workers – including Efrén Álvarez-Salvado as first author – shed new light on the rules that govern chemotaxis in flies walking in laminar flow and also explore what happens in more turbulent flow conditions.

Key to their effort was the development of small rectangular wind tunnels in which a constant laminar flow can be maintained, in which the flies can be constrained to walk in one plane, and in which the concentration of odor pulses delivered from a point source at the upwind end of a tunnel can be monitored at multiple points along the tunnel. With multiple tunnels in parallel, Álvarez-Salvado et al. – who are based at NYU, Weill Cornell Medical College and the University of Colorado Boulder – were able to monitor large numbers of flies under similar experimental conditions. They mostly used genetically blinded flies to limit the influence of visual cues, but sighted flies showed similar (though sometimes weaker) responses.

In trials with no odors, the flies tended to spend most of their time in the downwind end of the tunnel. However, when they first detected an odor pulse they usually responded by rapidly turning upwind, increasing their upwind velocity and decreasing the probability of turning: this is called the ON response. And after the odor pulse had passed, the flies decreased their upwind velocity and increased their turn probability, which resulted in a meandering local search for the source of the odor (Figure 1): this was the OFF response.

**Figure 1.**
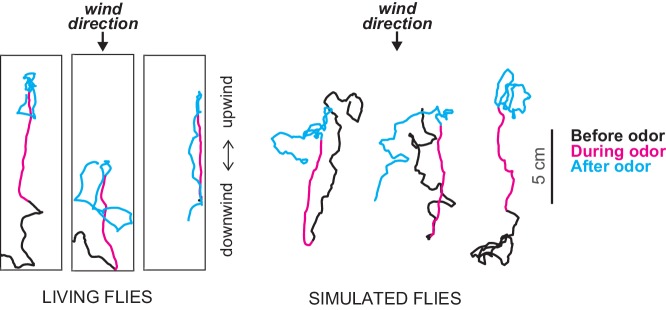
Exploring chemotaxis in flies in the presence of air currents. Álvarez-Salvado et al. performed experiments in which they tracked the movement of flies in small-scale wind tunnels when pulses of an odor (apple cider vinegar) were released from a point source (not shown) and were carried by the wind through the tunnel: in the figure the wind is blowing from top to bottom. Left: tracks of three genetically blinded flies before (black), during (magenta) and after (cyan) a 10 second odor pulse. At the onset of the pulse the flies turn rapidly toward the source of the odor and increase their upwind velocity. When the pulse ends the flies start to change direction in an effort to locate the odor. Right: based on these findings Álvarez-Salvado et al. built a model to simulate the movement of flies in the wind tunnels. The model does a good job of capturing the responses of the flies to various odor pulses.

Álvarez-Salvado et al. then showed that both the ON response and the OFF response depended on the odor concentration in a sigmoidal manner: that is, each response started to change at a particular concentration, continued to change as the concentration increased, and then stopped changing when the concentration reached a saturation value. The researchers were also able to make flies 'blind' to the wind by applying glue to their antennae: these files walked in all directions – not just upwind – in response to odor pulses.

The researchers concluded that, under their experimental conditions, the ON response is multi-modal, with changes in speed and turn probability depending on the odor concentration, and changes in orientation depending on wind direction (as detected by the mechanoreceptors on the antennae of the flies). The OFF response, on the other hand, is elicited by decreasing odor concentration.

These conclusions were corroborated and extended in experiments with more complicated odor pulses (including, for example, pulses in which the odor concentration fluctuated at different frequencies). A number of findings emerged from these experiments. First, the researchers observed a sensitivity adaptation that allows the fly to respond to a small decrease from a saturating concentration of odor much like *Drosophila* larvae in a stable odor gradient. Second, it appeared that the ON and OFF responses can be driven at the same time. Third, the response of the flies to fluctuations in the odor concentration depended on the frequency of the fluctuations. Fourth, the ON and OFF responses depended on the history of the odor in different ways, with rapid fluctuations (or a prolonged odor) leading to sustained ON responses and suppressed OFF responses.

Álvarez-Salvado et al. then tested these ideas in three interesting ways. First, they constructed simple models that embody these rules and used them to simulate how a fly in a wind tunnel responded to various odor pulses. The model produced realistic responses for both normal blind flies (Figure 1) and wind-blind flies. Second, they tested this model further by taking advantage of behavioral variability (that is, the fact that all flies will have slightly different responses to the same odor pulses). By scaling the ON and OFF response functions to individual flies, they were able to capture their individual responses and generate individually realistic tracks.

Third, Álvarez-Salvado et al. performed experiments in bigger wind tunnels in which the air flow was turbulent rather than laminar; the flies were also constrained to walk on the bottom surface of the wind tunnel. In the absence of odor, flies tended to move to the downwind end of the tunnel. When apple cider vinegar was introduced into the air stream, the flies responded by moving upwind, and performed local searches if they left the odor plume. While this behavior was similar to that seen in laminar flows in the small wind tunnels, the flies were less successful in locating the source of the odor. Simulated flies showed similar tracks and success rates.

As a result of these studies, we are beginning to understand how terrestrial navigation can be guided by air-borne odors. Although a chemical odor is an inherently non-directional cue, it acquires a directional component that can be used for navigation if the direction of air flow can be determined. Moreover, these studies show that the process of navigation itself can be broken down into component responses. In the case of the walking fly, the ON response (which depends on both odor and air-flow detection) turns the animal into the wind and keeps it on a relatively straight track, whereas the OFF response (which depends on odor detection only) leads to a local search to relocate the odor. These simple rules can also guide navigation under more natural or turbulent conditions (as in larger wind tunnels).

The importance of having done these experiments in *Drosophila* is that powerful genetic, optogenetic and electrophysiological tools can now be deployed to explore the neuronal mechanisms underlying the ON and OFF responses and their integration with the motor system that governs locomotion.

Keeping these cider vinegar rules in mind might also prove helpful when you next encounter the odor of fast food: just turn crosswind and head straight to carry yourself away from temptation.
